# Frequency of errors in the transfer of treatment parameters from the treatment planning system to the oncology information system in a multi‐vendor environment

**DOI:** 10.1002/acm2.13868

**Published:** 2022-12-16

**Authors:** William P. Donahue, Emily Draeger, Dae Yup Han, Zhe Chen

**Affiliations:** ^1^ Department of Therapeutic Radiology Yale University School of Medicine New Haven Connecticut USA

**Keywords:** data transfer, initial plan check

## Abstract

**Background:**

Technological advancements have made it possible to improve patient outcomes in radiotherapy, sparing both normal tissues and increasing tumour control. However, these advancements have resulted in an increase in the number of software systems used, which each require data inputs to function. For institutions with multiple vendors for their treatment planning systems and oncology information systems, the transfer of data between them is potentially error prone and can lead to treatment errors.

**Purpose:**

The goal of this work was to determine the frequency of errors in data transfers between the Varian Eclipse treatment planning system and the Elekta Mosaiq oncology information system.

**Methods:**

An in‐house program was used to quantify the number of errors for 2700 unique plans over an 8‐month period. Using this information, the frequency of the errors were calculated. A risk priority number was calculated using the calculated frequencies to determine the impact on the clinic.

**Results:**

The most common errors discovered were backup timer settings (10.7%), Field label (8.5%), DRR associations (3.3%), imaging field types (3.1%), dose rate (1%), Field Id (0.8%), imaging isocenter (0.7% and SSD (0.7%). Based on the risk priority numbers, the DRR association error was ranked as having the highest potential impact on the patient.

**Conclusions:**

The results of the work show that the most effort should be focused on checking the manual steps performed in the transfer process, while items that are imported directly from DICOM‐RT without modification are highly likely to be transferred accurately. The data can be used to help guide the implementation of future automated tools and process improvement in the clinic.

## 1 INTRODUCTION

Technological advancements have made it possible to improve patient outcomes in radiotherapy, sparing both normal tissues and increasing tumour control. However, these advancements have resulted in an increase in the number of software systems used, which each require data inputs to function.^1^ This transfer of data between systems carries an inherent risk of data entry errors or corruption but those involving the transfer of the treatment plan from the treatment planning system (TPS) to the oncology information system (OIS) can directly impact the patient treatment plan delivered from the linear accelerator (LINAC).^1^, ^2^ Integrated treatment planning and record and verify systems, e.g., Eclipse TPS and Aria OIS, do not require these types of transfers,^1^ however, many institutions use systems from multiple vendors, e.g., Eclipse TPS and Mosaiq OIS.^2^ For centers transferring treatment plans between the systems in a multi‐vendor environment, it is critically important that the fidelity of all plan parameters are checked beforehand to ensure the treatment is delivered as planned.^2^ However, the number of individual parameters to be checked during patient‐specific quality assurance (QA) can quickly become overwhelming due to treatment plan complexity.

AAPM Task Group 275 (TG‐275) recommended an extensive list of items that should be checked in a multi‐vendor environment.^2^ While some of these items are simple to check, for example, MU values for each field, others such as the control point leaf positions are infeasible to check for every control point in IMRT plans. The report discussed the importance of automation in chart checking, with a focus on providing an objective, efficient and standardized checking process.^2^ It also discussed some automated tools that have been created to streamline these checks.^3^, ^4^, ^5^, ^6^, ^7^ However, until these tools become widely available, clinical physicists need to determine the likelihood that these data errors may occur in order to prioritize their QA efforts. A single study by Rassiah et al.^8^ has performed a failure modes and effect analysis (FMEA) that included the transfer process between Eclipse and Mosaiq. While their FMEA focused on the entire treatment planning process, 63% of the failure modes identified were related to the treatment plan transfer and preparation in Mosaiq. The analysis suggested that the observable rates of these errors was less than 0.2% for all plans reviewed at the time of physics chart check. Both TG‐275^2^ and Rassiah et al.^8^ provide a coarse analysis of the data transfer between the systems in multi‐vendor clinics. However, when designing an efficient initial chart check process, more granular error frequencies may be required, especially when deciding where to focus efforts when implementing automation.

The goal of this work was to quantify the frequency of data transfer errors in a clinic that uses the Eclipse TPS and Mosaiq OIS combination. We calculated the error frequency using the logs of an in‐house program that checked the integrity of the plan data transfer. Using the calculated error frequencies, we performed a risk priority number (RPN) calculation for each type of error to determine its potential impact to the patient and clinical workflow.

## 2 METHODS

### 2.1 Clinic overview

The radiation oncology department consists of six clinics across the healthcare system with 11 linear accelerators (three Varian TrueBeam, three Elekta Synergy, and five Varian Trilogy). All clinics utilize the Eclipse Treatment Planning System (Varian Medical Systems, v16.1) and the Mosaiq Oncology Information System (Elekta Inc., v2.84) for record and verify.

#### 2.1.1 Plan transfer procedure

To provide a context for the errors observed in the clinical data transfer, a short overview of the data‐transfer process used in the department is provided.

1. Physician approves treatment plan in the TPS
2. Dosimetrist finalizes plan items, including
  - Field Labels and Names
  - Creating DRRs
3. Dosimetrist exports DICOM‐RT Plan and DRR files to Mosaiq
4. If a CBCT is used for localization
  - A copy of the plan is generated with a structure set containing only contours used for localization (this is performed to enhance the image review speed in Mosaiq)
  - This structure set and CT reference are exported to Mosaiq
5. Dosimetrist imports (promotes) plan in Mosaiq
6. Dosimetrist modifies plan parameters to ensure consistency and completeness
  - Field IDs corrected to fit OIS format
  - Field Names adjusted to correct format
  - Imaging modalities for Setup Fields selected
  - DRRs manually associated with all fields
  - SSD entered manually if missing from fields
  - CBCT Isocenter entered if missing from import
  - Dose rate changed to 0 for Elekta Linacs
  - Backup timer information entered for Varian Linacs
  - Tolerance Table set for treatment
  - Field dose parameters adjusted to handle rounding errors
7. Dosimetrist generates and uploads plan document to Mosaiq
8. Dosimetrist ensures prescription parameters (beam energy, fractionation, total dose, normalization values) match treatment plan, edits if necessary
9. Physician reviews and approves final prescription and treatment plan document

Once this process is completed the plan is ready for the chart checks. The chart checks in our institution include an independent dosimetrist check and a physicist chart check, which were completed manually prior to the introduction of the software discussed below. During the chart checks, both the dosimetrist and physicist verify that the treatment parameters were transferred accurately.

### 2.2 Automated data comparison software

**FIGURE 1:**
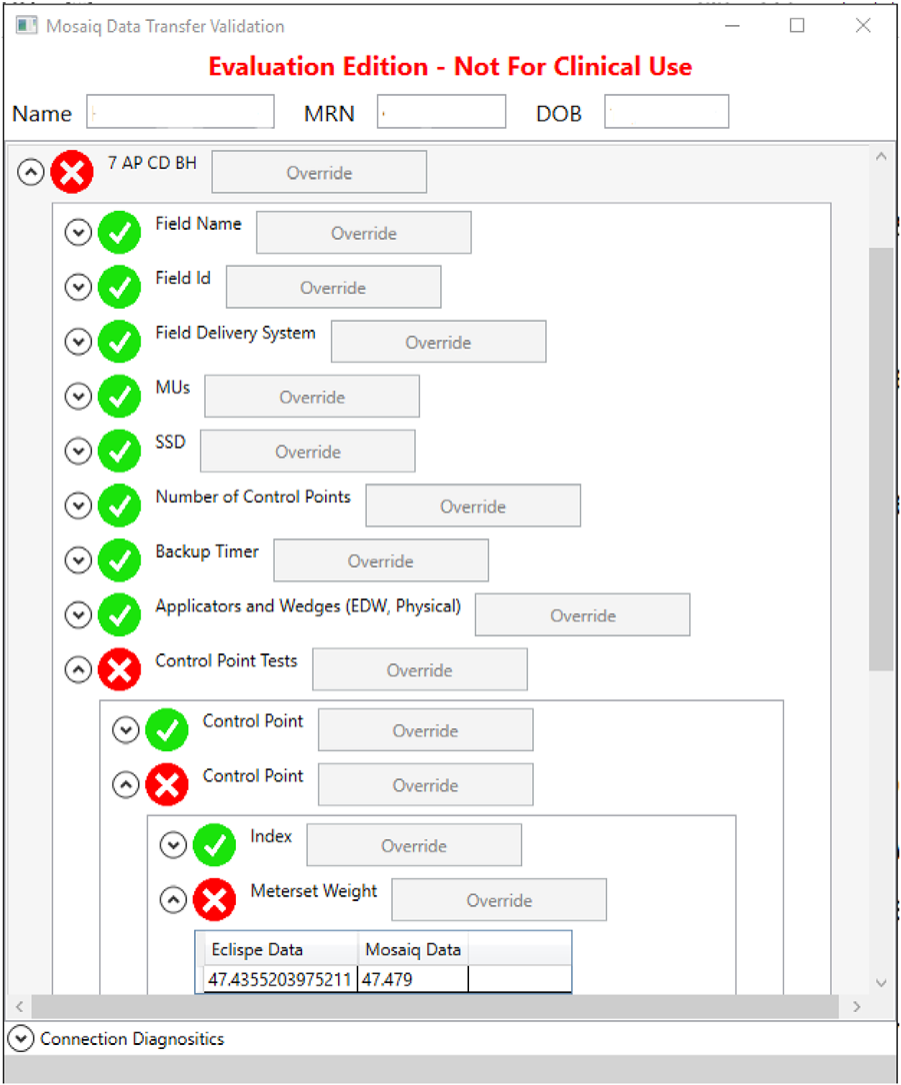
The graphical user interface of the E‐MTV showing the data for a treatment field. Green checks demonstrate items that passed the corresponding tests, while red X's show failures. The data presented shows an example error caused by rounding in the Meterset weights of a field using a motorized wedge on an Elekta Linac.

An in‐house program was developed to verify the data transfer process between Eclipse and Mosaiq. The Eclipse‐Mosaiq Transfer Validator (E‐MTV) compared treatment parameters stored in the Eclipse and Mosaiq databases. The program utilized the Eclipse Scripting API (ESAPI) to extract data from the database and provide user‐friendly integration into Eclipse. To extract the matching data from the Mosaiq database, the E‐MTV used SQL queries. The E‐MTV then compared the data and displayed it is an intuitive graphical user interface that allowed the user to review the test results and see the parameters compared (Figure 1). Finally, the tool stored the tested data in a file for error analysis and documentation of the tool's use. All of the parameters evaluated are listed in Table 1.

**TABLE 1:** This table shows the calculated RPN scores for each error mode evaluated by the E‐MTV

	Occurrence rate	O	S	D	RPN
DRR association	3.345%	9	6.0	4.5	243.0
CBCT/imaging isocenter	0.719%	7	6.9	3.8	179.8
Backup timer (min)	10.707%	10	3.8	4.3	159.4
Field name	8.428%	10	3.2	4.2	136.3
Dose per fraction (cGy)	0.396%	6	8.0	2.8	134.4
Number of fractions	0.252%	6	8.5	2.6	130.1
Applicators and wedges	0.124%	5	7.5	3.3	121.9
SSD (cm)	0.711%	7	4.8	3.3	108.1
First name	0.192%	5	8.5	2.5	104.9
Isocenter	0.077%	3	8.5	3.8	96.0
Field type (technique/imaging type)	3.128%	9	2.3	3.3	65.8
Field Id	0.787%	7	1.8	4.0	49.0
Control point Meterset weight (%)	0.001%	1	6.3	7.5	46.9
Dose rate	0.975%	7	2.3	2.8	43.3
Leaf positions	0.000%	1	6.0	6.8	40.5
Number of control points	0.000%	1	7.0	5.8	40.3
Jaw positions	0.000%	1	5.8	5.5	31.6
Field delivery system	0.033%	2	4.8	2.8	26.1
MRN	0.000%	1	8.5	2.7	23.1
last name	0.000%	1	8.5	2.5	21.0
Energy	0.000%	1	5.8	3.0	17.3
Couch angle (deg)	0.000%	1	6.8	2.5	16.9
Mus	0.000%	1	6.5	2.5	16.3
Gantry angle (deg)	0.001%	1	6.3	2.5	15.6
Collimator angle (deg)	0.001%	1	6.3	2.5	15.6

The Occurrence (O), Severity (S), and Detectability (D) scores were set using the scale from the TG‐100 report. Severity and Detectability were determined from the average score of the authors’ independent assessment.

The Eclipse and Mosaiq databases use difference schema to store the data. For example, Field IDs in Eclipse are 16 characters and are used at our institution to convey beam direction, site, and beam number. The Mosaiq field ID is meant only for the beam number and is five characters long. Another example is the use of double precision in Eclipse, while Mosaiq uses integer and decimal types with fixed precision values. This prevented using pure equalities to evaluate the treatment parameters. Instead, manipulations needed to be performed on both datasets. For string values, such as the field IDs, the program transformed the Eclipse values into a Mosaiq compatible format. For numerical values, it was necessary to implement a tolerance value for each test item performed. This value was selected to account for the rounding errors that occurred between the conversion from Eclipse to DICOM‐RT and then again from DICOM‐RT to Mosaiq and minimize the number of false negatives seen in the clinic.

### 2.3 Data analysis

Data log files were collected from an 8‐month period following initial testing of the E‐MTV (August 2021–April 2022). This resulted in 2774 unique plans being transferred from Eclipse to Mosaiq on which the tool was used. For each test performed by the E‐MTV, the detected error rate was calculated based on the first time the tool was run for each plan.

Using the analysis above, an RPN score was calculated for each test based on the TG‐100 framework. The observability, score was assigned using the scale presented in TG‐100 using the error rates seen in the clinic. The severity and detectability scores were determined by either a consensus of the authors or using the values for related items from TG‐275.^2^ Detectability scores were determined using the assumption that an automated tool was not available.

## 3 RESULTS

**FIGURE 2:**
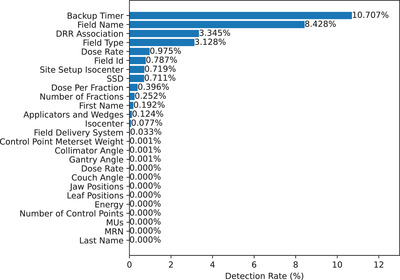
Detected rate of errors for each individual parameter tested by E‐MTV.

Of the 2774 plans evaluated, 574 (19.7%) had at least one failure detected by the E‐MTV. Inconsistencies between the field name or field id were found in 44.5% of the plans with a failure. The E‐MTV checked a total of 65.8 million individual parameters including the individual leaf positions of each control point. Overall, there was a 0.004% chance that any single parameter would have a failure. Figure 2 shows the rate of detected errors for each parameter for the first analysis performed using the E‐MTV. Of the 25 item categories checked by the tool, 11 had no errors detected, 12 had error rates less than 1%, and four had error rates greater than 2%. All detected errors were reviewed and resolved prior to each patient's treatment.

The top eight most common errors were, backup timer settings, field name inconsistencies, DRR association errors, imaging field type, dose rate settings, field id inconsistencies, site setup isocenter coordinate mismatches, and field SSDs. Each of these errors required manual entry or manipulation when importing the data into the Mosaiq OIS. The high incidence of certain errors, for example, backup timer and field name mismatches, were related to inconsistent applications of department standards for the data entry process. As an example, some team members preferred to use more detailed field names in Mosaiq where there are less character limitations. The most common error was the backup timer value inconsistency. Further review showed that the manually entered timer value in Mosaiq that was greater than the Eclipse calculated value, indicating that the fields would complete delivery but would have a potentially excessive safety margin. The DRR association errors were primarily caused by the need to manually assign and approve the images for use in Mosaiq. The field type errors were all related to the manual selection of the imaging modality in Mosaiq primarily for MV imaging fields. DRR association and field type errors were typically caught and corrected during the chart check, however, they occasionally made it to the patient's virtual simulation which led to patient treatment delays.

Table 1 shows an RPN analysis for each error type measured by the E‐MTV. These results show that, even when accounting for the Severity (S) and Detectability (D), the manual entry steps are the most important to review. Additionally, many of the potentially severe errors, for example, incorrect control point weights, gantry/collimator angles, and patient identification information, have a much lower RPN due to the robust implementation of the DICOM‐RT export and import protocols of Eclipse and Mosaiq, respectively. Based on this data, the clinic should focus on improving the standardization of the manual data entry process and add more attention in the chart checking procedure to the manual data components. This data also suggests that less focus may need to be paid on the control point and field‐specific parameters reviewed in this study.

## 4 DISCUSSION

We evaluated the relative occurrence of data transfer errors between the Eclipse TPS and the Mosaiq OIS using data from an in‐house tool. Using these error rates, we calculated risk priority numbers for each error type to improve the chart check effectiveness at our institution. The major finding was that the manual data entry steps required when transferring plans are the most prone to error, with the top eight most observed error types each involving a manual step. The DRR association was most significant for potential error due to the high frequency of incomplete or mismatched DRRs.

The implication of this work is that chart checks should focus on manual entry items when using mixed vendor solutions for the TPS and OIS. This implies that automated chart check solutions for the data transfer process should start by implementing checks on the manual entry, even though these may be the more difficult to implement. The data generated for this report provides a potentially useful baseline for the optimization of the chart check workflow.

Our data differs with most of the earlier literature on the errors in data transfer between the TPS and OIS. AAPM TG‐275 lumped the data transfer errors into a single failure mode category, with an observability score of 4.4^2^. In our study, we had an average observability score of 4.2 from all the error modes. However, our analysis revealed observability scores of up to 10 for some error modes which can be hidden when looking at the data in aggregate. The report by Rassiah et al.,^8^ reported an FMEA of their data transfer process between Eclipse and Mosaiq based on tabulation of manual chart check detected errors. Their analysis overlapped with ours in seven specific categories, that is, dose rate, field energy, field id, imaging field parameters, SSD missing, incomplete field data, and DRR association. A comparison of the observability and RPN scores are provided in Table 2. Our observability scores are on average 3.1 times higher than those reported by Rassiah et al.^8^ This disagreement could be related to the differences in clinical workflow and procedures between the institutions and the statistical uncertainty in both datasets. The RPN scores calculated in this work were higher for all but one item (field energy). This difference is most likely due to our assumption that the chart check was the only step in the process that checked this information, thus inflating our detectability and severity scores.

**TABLE 2:** Comparison of Observability (O) score and RPN for comparable failure modes in this work and Rassiah et al.^8^

	O			RPN		
	This work	Rassiah et al.	Ratio	This work	Rassiah et al.	Ratio
DRR association	9	3.2	2.8	243.0	13.1	18.6
CBCT/Imaging isocenter	7	2.3	3.0	179.8	40.3	4.5
SSD (cm)	7	1.0	7.0	108.1	4.1	26.5
Field type (technique or imaging type)	9	2.8	3.2	65.8	17.5	3.8
Field id	7	2.4	2.9	49.0	6.7	7.3
Dose rate	7	2.8	2.5	43.3	8.1	5.4
Energy	1	2.6	0.4	17.3	32.4	0.5

The major strength of this work is the ability of using the in‐house tool to collect the data from a variety of clinical situations during plan transfer. Due to the automated nature of the logs, the data was collected without requiring additional documentation by the staff. This allowed us to collect over 2500 unique plans for evaluation, which is significantly higher than previous studies.^3^, ^4^, ^5^, ^6^, ^7^, ^8^ Another strength is the scope of the plans reviewed. The plans encompassed a variety of treatment techniques, that is, 3D, VMAT, and SBRT, and treatment sites, for example, Breast, Lung, Prostate, Brain, Spine, etc. It also included plans created and transferred by 20 different dosimetrists stationed in six facilities across the healthcare system with both Varian and Elekta Linacs. This allowed the study to provide a better aggregate of errors across these different configurations.

This study is not without its weaknesses. First, the study was performed in a retrospective manner limiting our ability to control when the E‐MTV was run for each plan, as well as document and investigate the errors in a systematic way for this study. Future work is ongoing to address these uncertainties. The second limitation is that it is a single institution study. The E‐MTV was programmed to enforce institution policies for different parameters. Due to the continued standardization process being performed at our institution, the occurrence rates may be overestimated due to the interpretation of the policies by different team members. While the data presented in this work could provide a starting point, other institutions should validate these results to ensure their error rates are consistent. Finally, the E‐MTV received updates and improvements during the study period. This led to changes in error detection rates as the algorithm improved causing added uncertainty to the measured occurrence rates.

## 5 CONCLUSION

When designing a chart check program, it is important to prioritize the most hazardous and common errors to ensure an efficient and effective process. The data provided in this work quantified the frequency of different data transfer errors between the Eclipse TPS and Mosaiq OIS. The results of the work show that the most effort should be placed on the manual steps performed in the transfer process, while items that are imported directly from DICOM‐RT without modification are highly likely to be transferred accurately. This work is one of the first to quantify the error rates on MLC leaf position and other control point specific data transfer errors for the first time on a wide variety of plans, which would be infeasible to check manually. These results can be used to help guide future failure mode and effects analyses with respect to the data transfer between clinical systems.

## AUTHOR CONTRIBUTION

All authors contributed to the intellectual content of this work. William Donahue performed the data analysis and led the writing of the manuscript. Emily Draeger and Dae Yup Han assisted in the data collection process and provided revisions to the manuscript. Zhe Chen provided guidance and resources for the research project and was involved in the editing of the manuscript.

## CONFLICT OF INTEREST

The authors declare no conflict of interest
